# Common mechanisms involved in Alzheimer's disease and type 2 diabetes: a key role of chronic bacterial infection and inflammation

**DOI:** 10.18632/aging.100921

**Published:** 2016-03-09

**Authors:** Judith Miklossy, Patrick L. McGeer

**Affiliations:** ^1^ International Alzheimer Research Centre, Prevention Alzheimer International Foundation, Martigny-Croix, Switzerland; ^2^ Kinsmen Laboratory of Neurological Research, The University of British Columbia, Vancouver, B.C, Canada

**Keywords:** Alzheimer's disease, amyloid, Chlamydia pneumoniae, Diabetes type 2, Helicobacter pylori, infection, inflammation, spirochetes

## Abstract

Strong epidemiologic evidence and common molecular mechanisms support an association between Alzheimer's disease (AD) and type 2-diabetes. Local inflammation and amyloidosis occur in both diseases and are associated with periodontitis and various infectious agents. This article reviews the evidence for the presence of local inflammation and bacteria in type 2 diabetes and discusses host pathogen interactions in chronic inflammatory disorders. *Chlamydophyla pneumoniae, Helicobacter pylori* and spirochetes are demonstrated in association with dementia and brain lesions in AD and islet lesions in type 2 diabetes. The presence of pathogens in host tissues activates immune responses through Toll-like receptor signaling pathways. Evasion of pathogens from complement-mediated attack results in persistent infection, inflammation and amyloidosis. Amyloid beta and the pancreatic amyloid called amylin bind to lipid bilayers and produce Ca(2+) influx and bacteriolysis. Similarly to AD, accumulation of amylin deposits in type 2 diabetes may result from an innate immune response to chronic bacterial infections, which are known to be associated with amyloidosis. Further research based on an infectious origin of both AD and type 2 diabetes may lead to novel treatment strategies.

## INTRODUCTION

There is a strong association between Alzheimer's disease (AD) and type-2 diabetes [1-3]. Patients with type 2-diabetes have twice the risk of controls to develop AD [4-9], and the percentage of type 2-diabetes among AD patients is significantly higher than among age-matched non-AD controls [4, 10].

Insulin receptor (IR) and insulin-like growth factor-1 receptor (IGF-1R) are abundant in the normal human brain and are significantly decreased in AD. Insulin receptor substrate-1 (IRS-1) binds not only to IR and IGF-1R but also to the amyloid precursor protein (APP). Moreover, insulin-degrading enzyme (IDE) not only degrades insulin, IDE also degrades APP, amyloid beta (Aβ) [11] and amylin [12]. Insulin and insulin-like growth factor (IGF-1) prevent amyloid formation by decreasing APP in AD [13].

Amyloidosis is a group of conditions of diverse etiologies characterized by the accumulation of insoluble fibrillar proteins in various organs. Amyloidosis is a key pathological feature of both AD and type 2-diabetes, and chronic bacterial infections are frequently associated with amyloidosis. Bacteria and their toxic components, the bacterial endotoxin lipopolysaccharide (LPS) and bacterial cell wall peptidoglycan (BPG) are amyloidogenic and have been successfully used for almost a century in models of experimental inflammation and amyloidosis.

Because of the pioneering work of Warren and Marshall [14] infection with *Helicobacter pylori* (*H. pylori*) is accepted as a cause of gastro-duodenal ulcers. Recent observations show that infectious agents are factors in various chronic inflammatory disorders, including atherosclerosis, cardiovascular and cerebrovascular disorders [15-23], chronic lung diseases, [24-26], inflammatory bowel diseases, diabetes [27] and neuropsychiatric disorders, including AD [28, 29]. Polymorphisms in inflammatory genes are also implicated as risk factors in these age-related chronic disorders [30-32].

Increasing evidence supports an association between periodontal and systemic disorders [33, 34]. Periodontal pathogens are predominantly Gram-negative bacteria, and include *Porphyromonas gingivalis*, various oral Treponema (T) spirochetes *e.g. T. denticola, T. pectinovorum, T. amylovorum, T. maltophilum, T. medium and T. socranskii*, as well as various herpes viruses [21, 35-38]. Periodontitis is a risk factor for several chronic inflammatory disorders including atherosclerosis, stroke, diabetes and AD [33]. Aβ binds and disrupts lipid bilayers of bacterial membranes causing Ca(2+) influx and bacteriolysis [39]. Islet amyloid amylin is also able to form ion channels on lipid bilayers, causing Ca(2+) influx and cell destruction [40] and recent observations demonstrate antimicrobial activity of amylin [41, 42]. Thus, both Aβ and amylin accumulation are a response of the innate immune system to invading pathogens. This review considers recent information related to the role of local inflammation and involvement of pathogens in type 2 diabetes and AD.

**Figure 1:**
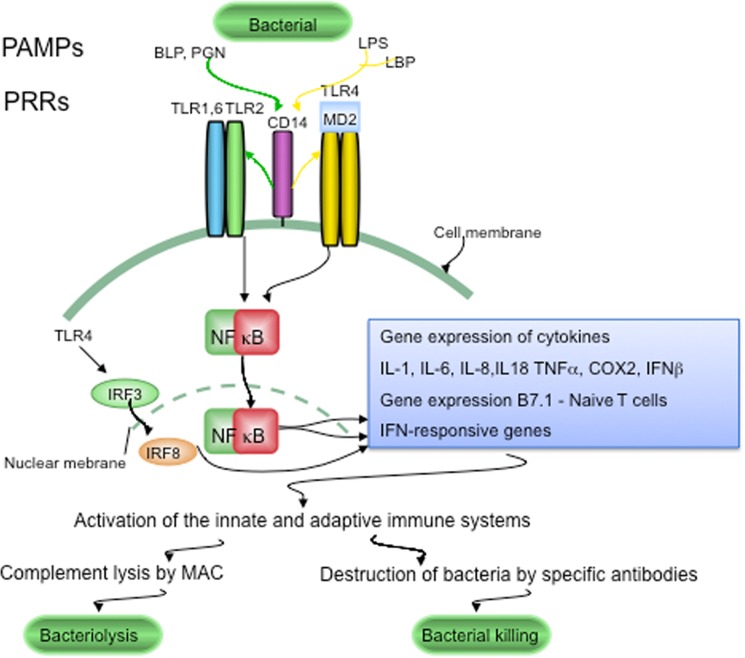
LTR signaling in host defenses against pathogens Conserved components unique to microorganisms, like bacterial lipopolysaccharide (LPS), bacterial peptidoglycan (PGN) and bacterial lipoproteins (BLP), are called pathogen-associated molecular patterns (PAMPs). PAMPs are sensed by pattern recognition receptors (PRRs), which include CD14 and various Toll-like receptors (TLRs). LPS is recognized following its binding to lipoprotein binding protein (LBP). CD14, is part of the LPS receptor complex, and together with the functionally linked TLR2 and TLR4, and the associated molecule MD-2 (lymphocyte antigen 96) are expressed in the endocrine cells of human pancreatic islets. PRRs and TLRs signaling pathways play a major role in maintaining pathogen-free host tissues. When TLRs are activated by PAMPs, through NFκB signaling the innate and adaptive immune systems are activated. Invading bacteria are killed by the terminal attack complex (MAC) of the classical complement pathway and by specific anti-bacterial antibodies provided by the adaptive immune system.

AD is characterized by a slowly progressive dementia and brain atrophy predominantly localized to the cerebral cortex and hippocampus. Senile plaques, and neurofibrillary tangles accumulate in parallel with disease progression. Senile plaques and tangles, argyrophilic filamentous structures, named curly fibers, neuropil threads or dystrophic neurites accumulate in abundance in the cerebral cortex [43, 44]. These slender curly structures are independent filaments, which do not form continuous networks. Their main length as determined by morphometric analysis is about 22 μm [44]. Amyloid deposits in AD mainly consist of Aβ, which include peptides of 40, 42 and 43 amino acid length, deriving from the larger 120 kDa APP by β- and γ-secretase cleavages [46-50]. APP is implicated in cellcell interactions, in regulation of immune system responses, and in T-cell differentiation [51-53]. Neurofibrillary tangles contain paired helical filaments composed mainly of the microtubule-associated protein tau, which is in a hyperphosphorylated state. The role of inflammation as a factor in the pathogenesis of AD is well established [32, 54-57]. McGeer, Rogers and Griffin documented first the importance of local inflammation and IL-1 signaling in AD [55, 58, 59]. Historic and recent observations indicate that pathogens are able to initiate and sustain chronic infection and inflammation and to reproduce the pathological and biological hallmarks of AD [29]. An analysis of the available historic and recent data following Koch's and Hill's criteria favors a causal relationship between spirochetal infection and AD [28]. Co-infection with several spirochetes also occurs in AD [28, 29, 60]. *Chlamydophyla (Chlamydia) pneumoniae* (*C. pneumoniae*), *Porphyromonas gingivalis, H. Pylori* and Herpes simplex virus type 1 (HSV-1) are other microorganisms, which are demonstrated in AD [61-64].

### Type 2 diabetes

Type 2 diabetes is a major health problem worldwide, and the number of AD cases is predicted to double during the next decades [65]. In order to solve this urgent problem, prompt action is recommended for both diseases [28, 66]. Type 2-diabetes, previously termed non-insulin dependent diabetes mellitus (NIDDM) or adult-onset diabetes, is characterized by progressive destruction of islet β-cells, resulting in decreased insulin production and decreased action of insulin on peripheral tissues. Type 2 diabetes is the most common form of diabetes, comprising 90-95% of all diabetic cases [67]. Local amyloid deposits are present in more than 95% of type 2 diabetic patients [68] and are mainly composed of amylin [69-71]. This islet amyloid polypeptide of 37 amino acids is derived by proteolytic cleavage of the 89-amino acid islet amyloid precursor protein or proamylin [69, 70, 72]. Amylin is related to the calcitonin/calcitonin gene-related peptide family [73]. Along with insulin, amylin is produced by β-cells in the Langerhans islets of the pancreas. The extent of amyloid deposition correlates with the clinical severity of diabetes, with the impairment in insulin secretion and glucose metabolism, and with the severity of beta-cell loss [69, 70, 74]. The cause of type 2 diabetes and the pathophysiological processes involved in amyloid formation are not well understood.

Aβ and insulin share a common sequence recognition motif [75], and Aβ is a direct competitive inhibitor of insulin binding and action, which reduces insulin receptor autophosphorylation [75]. Aβ and insulin are both substrates for the same insulin degrading enzyme (IDE). High-affinity interaction between Aβ and proamylin result in cross-suppression of cytotoxic selfassembly of both peptides, further suggesting a molecular association between AD and type 2 diabetes [76]. Beta-amyloid oligomers induce phosphorylation of tau and inactivation of insulin receptor substrate via c- Jun N-terminal kinase signaling [77]. It is noteworthy that insulin dysfunction can lead to tau phosphorylation *in vivo* [78].

### Aβ and tau in the pancreas in type 2 diabetes

Tau mRNA and protein expression are observed in normal and tumoral pancreatic and β-cell-derived cell lines [79-82]. Six predominant tau isoforms are identified by immunoblotting, which form tau deposits detectable by immunofluorescence and sarkosylinsoluble pellets [83]. The expression of APP and tau mRNAs by RT-PCR in normal and type 2 diabetes pancreas and in insulinoma beta cells (INS), as well as the detection of APP and tau immunoreactive bands by Western blot, indicates that APP and tau are present in the pancreatic tissue and in islet beta cells [82]. Slight upregulation of tau expression is also defined at the gene level in pancreatic islets in type 2 diabetic patients compared to normal age matched controls [84].

Aggregated Aβ, hyperphosphorylated tau, ubiquitin, apolipoprotein E, apolipoprotein(a), IB1/JIP-1 and JNK1 are immuno-expressed in the affected Langerhans islets in patients with type 2 diabetes [82, 85, 86] and Aβ is colocalized with amylin [82]. The secondary structure of islet amyloid deposits as analyzed *in situ* by Synchrotron InfraRed MicroSpectroscopy (SIMRS) reveals a protein (Amide I) absorbance maximum near 1630 cm-1, which is representative of beta-sheet protein structure and identical with the *in situ* infrared microspectra of amyloid deposits of senile plaques [82, 87, 88].

In transgenic mice carrying the carboxyl-terminus of the beta APP gene, Aβ is demonstrated not only in the brain, but also in the kidney and pancreas [89-91]. Formation of Aβ plaques is also observed in the pancreas in transgenic NORBA mice overexpressing human APP [92]. Remarkably, accumulation of Aβ and hyperphosphorylated tau also occur in brains of rat models of spontaneous diabetes, particularly of type 2 diabetes [93]. All these results indicate that Aβ formation and tau phosphorylation are also features of type 2 diabetes.

It is noteworthy that islet amyloid amylin is also observed in other organs than the pancreas, including the gut and kidney. Seventy-two of 149 patients with diabetic nephropathy showed amylin deposition in the kidney [94]. Amylin deposition in human brain and high affinity amylin binding sites in rat brains were also documented [95, 96].

### Local inflammation in type 2 diabetes

Systemic, rather than pancreatic inflammation, has been associated with type 2 diabetes since 1997 [97, 98]. Circulating markers of inflammation, acute-phase reactants and interleukin (IL)-6, the major cytokine mediator of acute-phase responses, are strong predictors of the development of type 2 diabetes [99-102]. Recent prospective studies have strengthened the association between type 2 diabetes and markers of systemic inflammation [103-110].

Only recently have studies focused on the possibility that, similarly to AD, local inflammatory processes might play an important role in type 2 diabetes. On haematoxylin and eosin-stained sections, discrete or moderate lymphocytic infiltrates were present in 2 of 8 diabetic cases analysed. Despite the apparent lack of lymphocytic infiltration in the remaining 6 diabetic cases, use of more sensitive markers revealed significant numbers of CD4 and CD 8 positive T lymphocytes in affected Langerhans islets [27]. Clumps of HLA-DR positive reactive macrophages accumulate around the islet amyloid deposits. Inflammatory cells expressing complement 3 receptor are also present in association with islet amyloid deposits and in the wall of some affected blood vessels [27]. This finding, together with abundant immunoreactivity to complement proteins C3d, C4d and C5b9 in association with islet lesions, indicates that the classical complement pathway is strongly activated in type 2 diabetes [27]. Recent observations demonstrate that both Aβ and amylin activate complement pathways [76]. These results show that, as in AD, local immune responses play an important role in the pathogenesis of type 2 diabetes [27]. In support of the importance of local inflammation in type 2 diabetes, an elevated number of immune cells are detected in the pancreatic islets in conjunction with increased levels of cytokines, chemokines, and IL-1 [111-114]. Animal models of type 2 diabetes investigated also display islet immune cell infiltration [114, 115].

**Figure 2:**
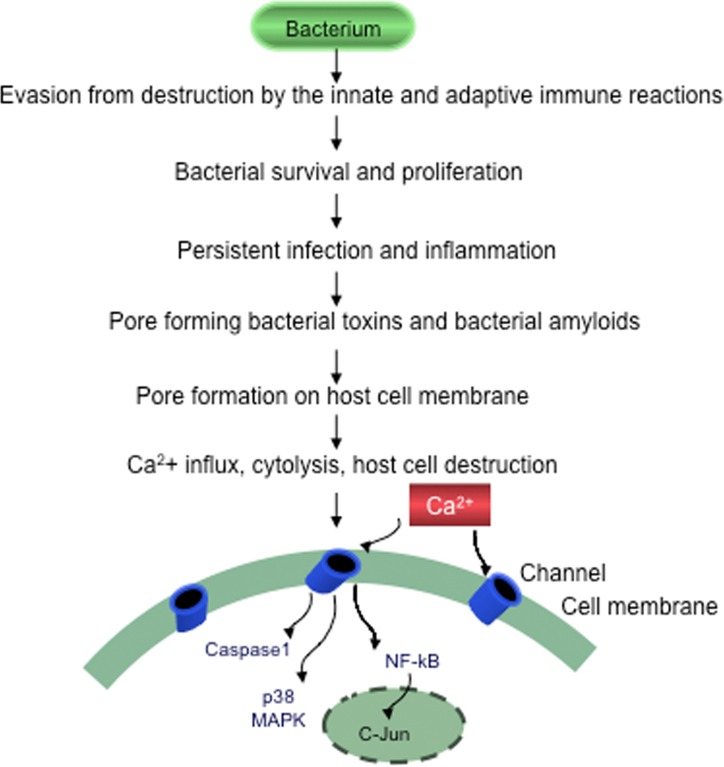
Evasion of bacteria from destruction by the host immune systems Bacteria by suppressing, subverting or escaping host defenses will survive, proliferate and cause persistent chronic infection, sustained inflammation and slowly progressive host cell destruction.

### Pathogens and type 2 diabetes

The onset of diabetes often occurs during or after an acute infection. Infections that lead to pancreatitis may produce diabetes [116]. Circulating LPS is higher in diabetic subjects compared to non-diabetics [117-119]. The association of diabetes with tuberculosis, syphilis and leprosy is well known [89, 90, 120-127]. It is noteworthy that various bacterial and viral infections, including *H. pylori,* enteroviruses, rubella, mumps, rotavirus, parvovirus and cytomegalovirus (CMV) have been proposed as potential etiological factors in type 1 diabetes [128-130].

An increased prevalence of *C. pneumoniae* IgA was observed in type 2 diabetes [131] and *C. pneumoniae* reinfection accelerated the development of insulin resistance and diabetes in obese C57BL/6 mice [132]. KKAy diabetic mice showed a significant increase in blood glucose, serum tumor necrosis factor-alpha (TNFα) and IL-6 levels after inoculation with the periodontal pathogen *Porphyromonas gingivalis* [133]. A high prevalence of *H. pylori* infection is observed in type 2 diabetes by many authors [134-145], with some exceptions [146-148]. A significantly higher percentage of positive fluorescent treponemal antibody reaction is observed among diabetic patients compared with nondiabetics [78, 149]. Diabetes mellitus is more prevalent among Borrelia-infected patients [150].

Periodontal disorders are risk factors for type 2 diabetes [151-157] and are highly prevalent, affecting up to 90% of the worldwide population [153]. Both type 1 and type 2 diabetic patients show a three- to four-fold increased risk of periodontitis [158-161]. Type 2 diabetic patients have significantly more severe periodontitis, a higher plaque index and a higher prevalence and magnitude of root surface caries than non-diabetic subjects. Periodontitis is predominantly caused by Gram-negative anaerobic bacteria [151-154] and several spirochetes are also demonstrated to be periodontal pathogens. Checkerboard DNA-DNA hybridization showed that significantly more diabetic subjects have higher levels of *T. denticola* compared to controls [162]. Treatment of periodontal infections improves glycemic control in diabetic patients [151-154, 163].

The association of periodontitis and diabetes suggests that microorganisms are pathogenic in type 2 diabetes [27, 152-154]. The presence of various bacteria associated with islet lesions in patients with type 2 diabetes is demonstrated pathologically [27]. Anti-core- LPS J5, anti-Lipid A and anti-BPG antibodies reveal positive LPS and BPG immune-reactions in association with islet amyloid deposits in the pancreas of 8 patients with clinically and pathologically confirmed type 2 diabetes [27]. On doubly immune-stained sections, LPS and BPG were immuno-co-localized with amylin, and LPS-positive and BPG-positive helically shaped, round, and fusiform bacteria are observed in the affected islets. Intracellularly located immune-reactive *C. pneumoniae* was observed in the affected Langerhans islets in 5 of 6 diabetic cases tested, and extracellularly located, slightly coiled *H. pylori* bacteria are demonstrated in 3 of these same diabetic patients. Spirochetes are also observed in the affected islets, explaining the high frequency of fluorescent Treponemal antibodies found in diabetes [27]. These bacterial structures are immunoreactive for C5b-9, the complement membrane attack complex (MAC) intended to lyse bacteria. As MAC assembles on cell membranes *in vivo,* the possibility is eliminated that C5b-9 immuno-reactive bacterial structures may correspond to post mortem bacterial growth. Moderate LPS and BPG immuno-reactivity was also observed in the pancreas of three controls in association with mild islet amyloid deposition, corresponding to pre-clinical stages of type 2 diabetes in these cases. The remaining 9 controls were negative [27].

In agreement with epidemiological studies, the presence of bacteria, including *C. pneumoniae, H. pylori* and various types spirochetes in pancreatic islets supports a pathogenic role of bacterial infection in type 2 diabetes. Simultaneous occurrence of various types of bacteria in the pancreatic islets of the same diabetic patient suggests that concurrent infection by several pathogens occurs in type 2 diabetes, as observed in atherosclerosis [21, 22] and AD [28, 29, 60-62, 64]. *C. pneumoniae* [15, 16], *H. pylori* [17, 18], several periodontal pathogens, including invasive oral spirochetes [19, 20] and herpes viruses, have also been demonstrated in human atherosclerotic lesions. Some microorganisms were shown to enhance atherosclerosis in experimental animals [20, 21].

Improvement of glycaemic control was observed after eradication of *H. pylori* infection in diabetic patients, suggesting that early antibiotic and anti-inflammatory treatment may be an effective way to prevent or slow down the disease process [164].

### Host pathogen interaction through toll-like receptors

Whole bacteria and specific microbial components (LPS, BPG) are demonstrated in islet lesions in type 2 diabetes [27, 118, 165]. Such conserved microbial motifs (LPS, BPG, various bacterial lipoproteins, bacterial DNA etc.) are called pathogen-associated molecular patterns (PAMPs). PAMPs are sensed by pattern recognition receptors (PRRs) [166], which trigger an immediate response against invading pathogens. The major forms of PRRs are Toll-like receptors (TLRs) and some nucleotide-binding oligomerization domain (NOD) receptors called Nodlike receptors (NLRs) [167]. Once TLRs and NLRs are activated by PAMPs through signaling pathways, they induce innate and adaptive immune responses [167, 168]. Thus signaling by TLRs and NLRs is a key component of immune responses to microbial infection [169].

CD14, which is part of the LPS receptor complex, together with the functionally linked TLR2 and TLR4, and the associated molecule MD-2, are all expressed in the endocrine cells of human pancreatic islets. SV40- transformed islet cells (HP62) synthesize and secrete CD14 in response to LPS in a time- and dose-dependent manner. *In vitro* experiments using rat islets, which also express CD14, as well as HP62 cells, showed that LPS influences glucose-dependent insulin secretion and induces formation of inflammatory cytokines such as IL-1α, IL-6 and TNFα [170]. LPS also induces increased APP and tau levels in neuronal and nonneuronal cells *in vitro* [82].

TLRs are involved in a variety of diseases including atherosclerosis, type 2 diabetes, liver disease, inflammatory bowel diseases and AD [165]. NLRs together with TLRs induce IL-1β and IL-18, which are important mediators in most inflammatory disorders [168]. Expression of various PAMPs and PRRs, including CD14 and TLRs, together with local immune responses in association with the lesions in both AD and type 2 diabetes, indicates that microorganisms and PAMPs induce and sustain chronic infection and inflammation in these chronic amyloidogenic disorders. The role of bacteria as sources of PAMPs via TLR2 and TLR4 stimulation, in atherosclerosis, type 2 diabetes, AD suggests a common pathogenic process in these diseases.

Antimicrobial peptides (AMPs) are another important group of molecules of the innate immune system, which combat invading microorganisms. Recent observations reveal that Aβ, the most important biological marker of AD, is an innate immune molecule, and shares properties with AMPs [39]. Soluble Aβ 1-42 oligomers form channels on lipid cell membranes and cause Ca(2+) influx and cell destruction [171]. Aβ at high doses exerts antimicrobial activity *in vivo* against eight common and clinically relevant microorganisms of the 12 tested. Antimicrobial activity of brain homogenates was attenuated by immune-depletion of Aβ [38]. CT105 peptide, a C terminal fragment of APP also forms ion channels or pores [172] and the microtubule binding sites of tau have been shown to harbor somewhat similar properties [173].

The level of human serum amyloid A (SAA), an acute phase protein, which rises during infection, also forms channels on lipid bilayer membranes with resulting Ca(2+) influx. Expression of a recombinant acute phase isoform variant of human SAA 1.1 (SAAp) induces bacteriolysis, suggesting an important role in host defenses [174]. It is noteworthy that amylin also forms ion channels on lipid bilayers with consequent Ca(2+) influx and cell lysis [40, 175]. Recent observations show that amylin is an antimicrobial peptide, which can augment host defenses against on-going infection, as also observed for Aβ [39] and SAA.

Permeabilization of lipid bilayers is a common conformation-dependent activity of soluble amyloid oligomers in various neurodegenerative disorders [176]. Blockade of an amyloid peptide channel by zinc, and inhibition by Congo red, has been recently reported [177].

It has been hypothesized that amyloid pores may in fact be beta-sheet barrels similar to pore forming bacterial toxins [178]. Pore-forming toxins are the most common class of bacterial protein toxins and are often important virulence factors. These toxins are typically oligomers of soluble, monomeric proteins or peptides, which form transmembrane channels. Channel formation in the membrane of targeted cells triggering cellular ion imbalance is a frequent form of bacterial attack [179, 180]. These pore-forming bacterial toxins generate calcium-dependent and lipid-mediated signaling on host cell surfaces, leading to a variety of events such as tyrosine phosphorylation [181], actin rearrangement [182], NF-κB activation [183] and regulation of gene expression through histone modification [184].

It is noteworthy that amyloid proteins constitute a previously overlooked integral part of the cellular envelope of many bacteria [185-189]. Amyloid fibril formation not only results in toxic aggregates, but also provides biologically functional molecules [186, 187, 190], which play a role in virulence, invasion and host cell destruction. Recent observations show that the amyloid oligomers associated with human diseases, perforin from cytotoxic T lymphocytes and poreforming bacterial toxins, share structural homology and the same mechanism of membrane permeabilization [191].

The amyloidogenic properties of perforin and the bacterial pore-forming toxin alpha-hemolysin were demonstrated spectroscopically and morphologically [191].

The observations on amyloidogenesis suggest that host cells and bacteria, during host-pathogen interactions, use similar molecular mechanisms to induce host cell lysis and bacteriolysis. Further studies will be required to determine whether host cell destruction predominates over bacteriolysis in chronic sustained infections and inflammations.

In addition, genetic predisposition of the host, the virulence and biology of the invading pathogens, and environmental factors, including nutrition and demographic conditions, are also key determinants of disease expression.

### Establishment of chronic infection, inflammation and progressive cell damage

During infection, pathogens employ a broad range of strategies to overcome antigenic recognition, phagocytosis and complement lysis. Blockade of the complement cascade allows their survival even in immune competent hosts. If pathogens are not recognized by the immune system, and in the absence of cell-mediated immune responses, the microorganism can spread freely and accumulate in affected host tissues [192]. Under such conditions, microorganisms will establish chronic infection, inflammation and progressive tissue damage. Host responses to bacterial infections are genetically controlled. Polymorphisms of proinflammatory cytokine genes are associated with susceptibility to infection [193]. TNF-α and Class II major histocompatibility genes are critical mediators of host defences against infection by influencing host immune responses to bacterial and viral infections. Polymorphisms in the gene encoding TNF-α may determine a strong cell-mediated immune response or a weak or absent cellular response [193, 194]. Human leukocyte antigen (HLA) also controls cell-mediated responses [195]. HLA-DR isotypes are associated with a protective response, whereas HLA-DQ isotypes have a more limited cellular response but with a higher number of microorganisms. Accordingly, a polarity in host reactions can be observed in various infections. In tuberculoid or paucibacillary leprosy, inflammatory cell infiltration is strong and the number of microorganisms is low. However in lepromatous or bacillary leprosy, the poor or absent inflammatory cell infiltrates are accompanied by a high number of *Mycobacterium leprae* bacilli. A similar polarity in host reactions also occurs in neurosyphilis. Strong cell-mediated immune responses and a low number of spirochetes characterize the infiltrative form of general paresis. In the atrophic form of general paresis, the lymphoplasmocytic infiltrates are poor or absent, but the number of spirochetes is high [196-199].

If infectious agents are involved in diabetes, one may expect that such polarities in host reactions might also be present. According to this view, type 1 diabetes is characterized by strong cell mediated immune response with a low number of microorganisms and type 2 diabetes is characterized by poor or absent lymphoplasmocytic infiltrates with high number of microorganisms. Further studies are needed to support this suggestion.

## DISCUSSION

Common cellular and molecular mechanisms are implicated in the pathogenesis of AD and type 2 diabetes. In addition to amylin, Aβ and hyperphosphorylated tau accumulations are also features of islet lesions in type 2 diabetes. Ubiquitin, Apo-E, Apo(a), IB1/JIP-1 and JNK-1 are all associated with brain lesions in AD and islet lesions in type 2 diabetes. As in AD, both cellular and humoral components of local immune responses are involved in the pathogenesis of type 2 diabetes, as indicated by the presence of PAMPs and PRRs in the affected Langerhans islets. The classical complement pathway is activated in both AD and type 2 diabetes. Using sensitive immuno-markers of B and T lymphocytes, increased numbers of CD4, and particularly of CD 8 positive T cells, are demonstrated in both AD and type 2 diabetes, indicating a minimal involvement of the adaptive immune system.

Epidemiological and pathological evidence demonstrate the prevalence of pathogens in several chronic inflammatory diseases, including atherosclerosis, AD and type 2 diabetes. An increased prevalence of *C. pneumoniae* IgA, *H. pylori* infection, and a significantly higher percentage of positive fluorescent treponemal antibody levels are observed among diabetic patients compared to controls, indicating the involvement of spirochetes in diabetes. These observations indicate that various types of spirochetes are also involved in diabetes. As AD, atherosclerosis and diabetes are all associated with periodontal disorders, to consider periodontal pathogens, including *Porphyromonas gingivalis* and various periodontal pathogen spirochetes and various intestinal bacteria, including intestinal spirochetes, in the etiology of these chronic inflammatory disorders is important. *Borrelia burgdorferi* and other Borrelias might also be candidate spirochetes in some cases with type 2 diabetes. The observation of several of these bacteria in association with islet lesions suggests that chronic bacterial infection could be directly involved in the pathogenesis of type 2 diabetes. Similarly to periodontitis, simultaneous infection with multiple pathogens also occurs in type 2 diabetes. Invading pathogens, and their persisting toxic PAMPs sensed by PRRs through TLRs signaling, induce innate and adaptive immune responses. In the affected pancreas, pathogenic bacteria and their toxic components can be observed, along with a host immunological reaction, which is characteristic of a localized inflammatory process associated with the sites of tissue damage, similar to the inflammatory process observed in AD [58, 59, 200, 201].

## CONCLUSION

Increasing evidence supports the hypothesis that bacteria or their slowly degradable remnants, may initiate a cascade of events leading to persistent chronic infection, β-cell loss and amyloid deposition in type 2 diabetes. Increased Aβ and amylin accumulation, and the resulting AD and islet pathology, may be mediated by a response of the innate immune system to infection. An infectious origin may give an explanation of the common pathogenic mechanisms and inflammatory gene polymorphisms involved in both AD and type 2 diabetes. This interpretation may have important implications for current and future treatment strategies, opening the possibility of preventing infection, inflammation and amyloidosis in both AD and type 2 diabetes.
